# Comparison of drug-eluting balloon versus drug-eluting stent for treatment of coronary artery disease: a meta-analysis of randomized controlled trials

**DOI:** 10.1186/s12872-018-0771-y

**Published:** 2018-03-02

**Authors:** Lulu Liu, Bin Liu, Jiajun Ren, Gang Hui, Chao Qi, Junnan Wang

**Affiliations:** Department of Cardiology, Second Hospital of Jilin University, No. 218 Ziqiang Street, Changchun, 130041 China

**Keywords:** Coronary artery disease, Drug-eluting balloon, Drug-eluting stent, Meta-analysis

## Abstract

**Background:**

Drug-eluting balloons (DEB) have significant value for treating coronary artery disease (CAD). However, the merits of using DEB versus drug-eluting stents (DES) to treat CAD remain controversial. Herein, we conducted a meta-analysis to compare the safety and efficacy of DEB and DES for treatment of CAD.

**Methods:**

We searched MEDLINE, EMBASE, and CENTRAL databases for eligible trials comparing DEB with DES for treatment of CAD. The primary endpoint was major adverse cardiac events (MACE); the secondary endpoints included in-lesion late lumen loss (LLL), binary restenosis (BR), myocardial infarction (MI), target lesion revascularization (TLR) and mortality.

**Results:**

Twenty-three trials with a total of 2712 patients were included. There were no significant differences in the primary endpoint of MACE between the DEB and DES groups (Risk Ratio (RR) 1.19; 95% confidence interval (CI) (0.87, 1.63); *P* = 0.27), or in the clinical outcomes of each of MACE’s components, including TLR, MI and mortality. However, efficacy was significantly different between the DEB and DES groups, especially when we compared DEB to second-generation DES: in-lesion LLL (Mean Difference (MD) 0.11; (0.01, 0.22); *P* = 0.03); binary restenosis (RR 1.46; (1.00, 2.13); *P* = 0.05).

**Conclusions:**

DEB is equivalent to DES in terms of safety for managing CAD, and DEB may be considered as an alternative choice for treatment of CAD.

**Electronic supplementary material:**

The online version of this article (10.1186/s12872-018-0771-y) contains supplementary material, which is available to authorized users.

## Background

Coronary interventional therapy has significantly improved the prognosis of patients with CAD [1, 2]. In particular, the clinical application of drug eluting stents (DES) has greatly reduced the occurrence rate of in-stent restenosis (ISR), which is one of the major complications associated with bare metal stents (BMS) [3]. However, there are still several DES-linked potential complications, such as delayed vascular endothelial healing, late and very late stent malapposition and stent thrombosis, and emerging atherosclerosis [4, 5]. In addition, DES is not effective at treating some complex coronary artery lesion subsets, such as small vessel disease (SVD) (defined as target vascular lesions at both ends of the reference diameter ≤ 2.75 mm) [6] and bifurcation [7], suggesting that DES is not optimal for certain CAD. Besides, diabetic patients with coronary artery stenosis have worse clinical outcomes including mortality and repeated revascularization which related to in-stent restenosis and stent thrombosis [8, 9]. Hence, it is imperative to devise a new treatment strategy which may offer effective treatment while reducing complications for those “difficult” CAD cases.

Based on the above-mentioned limitations of DES, DEB was designed to avoid insertion of a permanent foreign object in the blood vessel, and therefore prevent potential problems caused by DES [10]. DEBs are coated with a thin mixture of pharmacologically active, high dose anti-proliferative drugs on the base of an ordinary balloon. The drugs are transferred to the neointimal layer of the vascular wall upon a single inflation of the balloon, which typically takes a maximum of 30–60 s in coronary intervention [11–13]. To circumvent the elastic recoil that occurs after DEB inflation, a bare metal stent (BMS) is subsequently employed [14].

However, the superiority of either DES or DEB for treating coronary stenosis remains unknown. Therefore, we performed this meta-analysis to compare the advantages and disadvantages of DEB (in combination with BMS or as a stand-alone therapy) and DES in the treatment of CAD and systematically reviewed the safety and efficacy of DEB in clinical applications.

## Methods

### Search strategy

Two independent investigators (Lulu Liu and Jiajun Ren) searched the MEDLINE, EMBASE and CENTRAL databases. We also considered published review articles, editorials, and internet-based sources of information ((http://www.tctmd.com), (http://www.europcronline.com) and (http://www.crtonline.org)) to assess potential information on trials of interest. Conferences from the American Heart Association and American College of Cardiology were reviewed as well. The study was performed according to the Preferred Reporting Items for Systematic Reviews and Meta-analyses (PRISMA) guidelines for meta-analyses of randomized trials [15]. The search end date was September 4th, 2017. The search keywords were (“drug-eluting balloon” OR “drug coated balloon”) AND (“drug-eluting stent” OR “drug coated stent”) AND (“randomized controlled trial” OR “controlled clinical trial”) NOT (“popliteal” OR “femoropopliteal” OR “infrapopliteal” OR “infrainguinal”). The detailed search strategy is shown in Additional file 1. Trials were limited to human trials. The selection process was conducted with the titles and abstracts of all citations to identify potentially relevant trials. Then, the corresponding publications were reviewed in the full text to assess if trials met the inclusion criteria. The selection process and data extraction were completed by the two investigators independently. When there was no agreement between the two reviewers, a third person was involved to discuss the situation and make the final decision.

### Selection criteria and interest of endpoints

Trials that had all of the following were included in the analysis: (1) randomized controlled trials (RCT); (2) subjects in the study had CAD; and (3) the intervention measure of the study involved DEB and DES. Trials that had one or more of the following were excluded from the analysis: (1) non-RCT, such as observational trials or retrospective trials; (2) incomplete or having statistical differences of baseline data; and (3) no available full text. The primary endpoint was major adverse cardiac events (MACE). The secondary endpoints were in-lesion late lumen loss (LLL) (calculated as the difference of in-stent minimal lumen diameter (MLD) between measurements immediately after the procedure and at follow-up) [16], binary restenosis (BR) (defined as diameter stenosis > 50% by quantitative coronary angiography (QCA) at the follow-up angiogram) [17], and the components of MACE (myocardial infarction (MI), target lesion revascularization (TLR), and mortality). Clinical outcomes were considered safety outcomes, such as MACE and each of its components, whereas procedural outcomes, including LLL and BR, were considered efficacy outcomes.

### Data extraction and quality assessment

We extracted study information, such as baseline characteristic data, major endpoints, number of patients, type of disease, and other related information, from the selected trials, and then summarized the data in a prepared standardized extraction database (Microsoft EXCEL). We used the RCT Document Quality Evaluation Criteria recommended by the Cochrane Handbook 5.1.0 [18] to assess the quality of included trials based on the following points: sequence generation, allocation concealment, blinding of participants and personnel, blinding of outcome assessment, incomplete outcome data, selective outcome reporting and ‘other issues’. A study was judged using the labels ‘low risk’, ‘high risk’ or ‘unclear risk’.

### Statistical analysis

For comparison of the dichotomous and continuous data, risk ratio (RR) and mean difference (MD) and their 95% confidence intervals (CI) were used as the effect indicators. Mantel-Haenszel method was used for combining RRs, and the overall MD was built with the inverse variance method as recommended [19]. *P* ≤ 0.05 was considered statistically significant for overall effect. Statistical analysis was performed using the Review Manager 5.3 software (The Nordic Cochrane Centre, The Cochrane Collaboration, Copenhagen, Denmark). The heterogeneity of the included trials was analyzed by Chi-squared test and I^2^ statistic based on the Mantel-Haenszel random-effects model for combining RRs or Inverse Variance random-effects model for MD. The test level was set to α = 0.1, in combination with I^2^ to access the heterogeneity quantitatively, and I^2^ > 50% was regarded as being indicative of moderate-to-high heterogeneity [20]. A *P* value ≥0.1 suggested that there was no statistical heterogeneity among the results. We then used the fixed effect model for meta-analysis, and *P* < 0.1 suggested that there was statistical heterogeneity among the results. In this case, we used the random effect model for the meta-analysis [21, 22]. Analyses were carried out for overall coronary artery conditions and then stratified by the method of treatments and type of lesions, or on the basis of factors that may lead to heterogeneity, such as different generations of DES and follow-up length. Sensitivity analysis was performed by removing the results of individual trials one by one to observe the changes in effect size. We used Begger Funnel plot and Egger tests with Stata (version 12.0) to assess publication bias, with a *P* value < 0.05 was considered statistically significant.

## Results

**Fig. 1:**
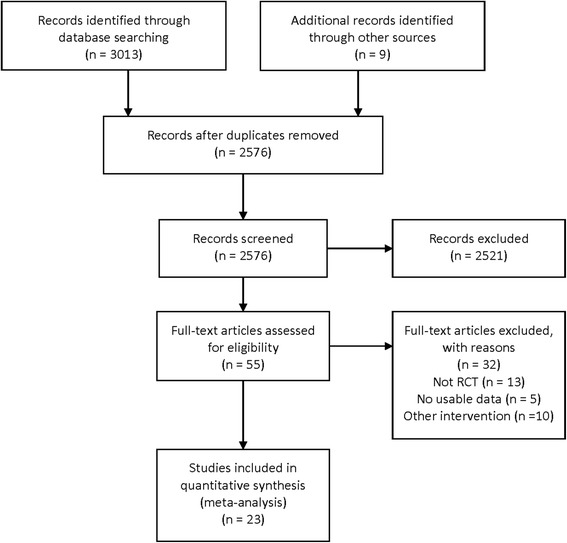
Search flow diagram

**Fig. 2:**
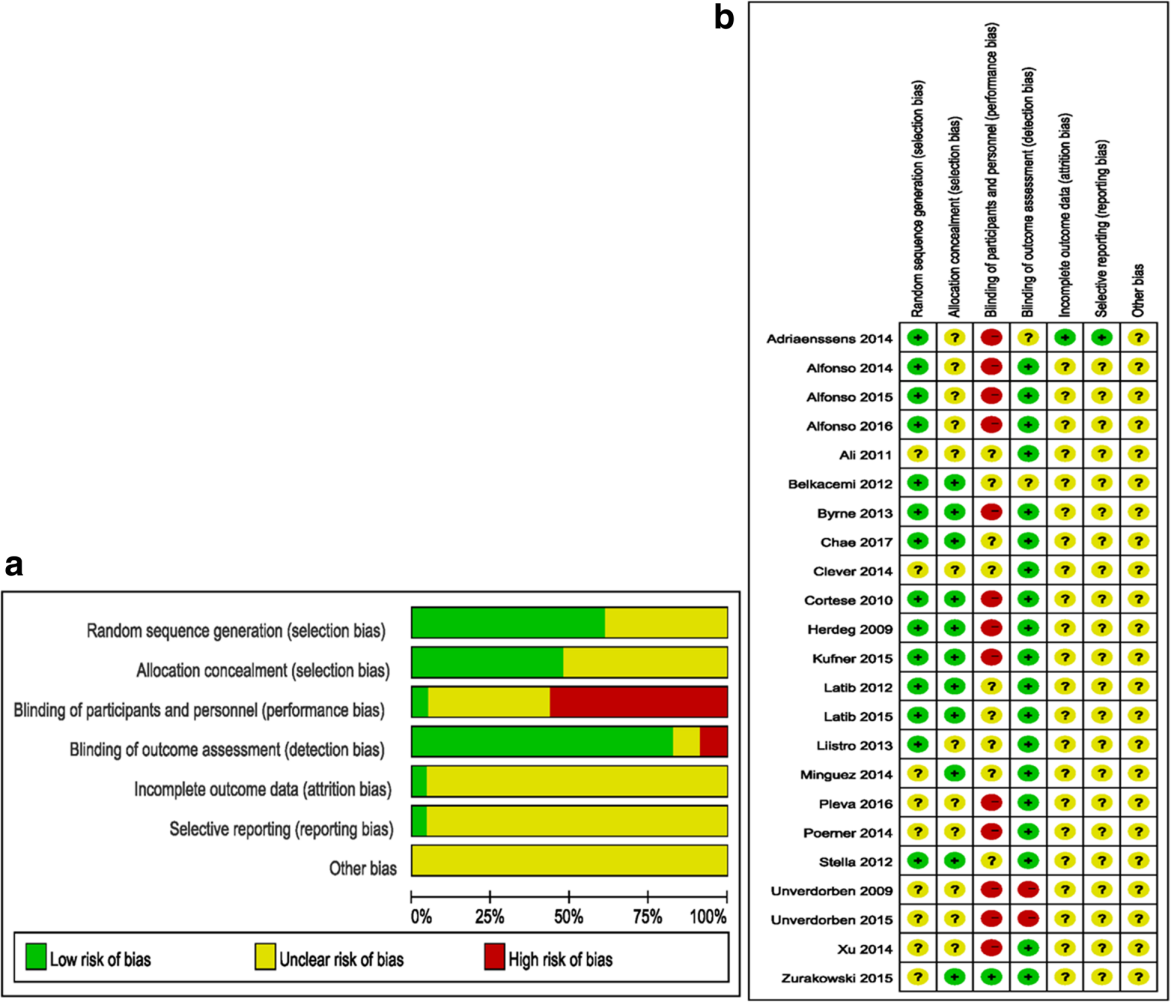
Risk of Bias Assessment. **a** Risk of bias graph; **b** Risk of bias summary

The study flow chart was established using PRISMA (Fig. 1). The initial search result collected 3013 publications. By excluding the repetition in the literature and applying the inclusion and exclusion criteria, 55 full text papers were reviewed and 23 articles were finally included, which included a total of 2712 patients [23–45]. The angiographic follow-up period was 6 to 12 months, and the clinical follow-up period ranged from 6- to 36-months. The types of CAD reported in the literature included seven simple coronary lesions [23–29], seven ISR [30–36], two bifurcated lesions [40, 41], two small vessel disease (SVD) [37, 38], and one diabetes-linked coronary artery stenosis [39]. All trials were RCT, four of which were 3-year follow-ups of PEPCAD II [42], BELLO [43], ISAR-DESIRE 3 [44], and RIBS V [45]. The study population included patients who were recruited from the beginning of the study without any abnormal clinical characteristics, and were only described the clinical data after 3 years of follow-up. We extracted the number of patients, the types of DEB and DES, follow-up time, dual anti platelet therapy (DAPT) duration, and other information needed for this analysis from each study. The baseline characteristic data between the DEB and DES groups are summarized in Table 1, and more baseline clinical data are summarized in Additional file 2. There were no significant differences between these two groups with regards to baseline characteristics of the patients. The risk of bias assessment of included trials is shown in Fig. 2.

**Table 1:** Baseline characteristics of included trials

Study	Disease	No. of patients	Type of devices		DAPT(mts)	MACE	Follow-up (mts)	Pre- dilatation (n/lesions)	
			DEB(+BMS)	DES				DEB	DES
Adriaenssens 2014 [30] SEDUCE	BMS-ISR	50	PEB(SeQuent Please)	EES(XIENCE V/Prime)	NA	NA	9 (angio), 12 (clinical)	25/25	25/25
Alfonso 2015 [31] RIBS IV	DES-ISR	309	PEB(SeQuent Please)	EES(XIENCE Prime)	3 after DEB, 12 after DES	NA	6–9 angio), 12–36 (clinical)	154/154	155/155
Alfonso 2014 [32] RIBS V	BMS-ISR	189	PEB(SeQuent Please)	EES(XIENCE Prime)	3 after DEB, 12 after DES	NA	6–9 (angio), 12 (clinical)	95/95	94/94
Ali 2011 [39] PEPCAD IV	coronary stenoses in patients with DM	84	DEB(SeQuent Please) + cobalt-chromium stent(Coroflex Blue)	DES (Taxus Liberté)	3 after DEB, 6 after DES	NA	9	14/45	38/39
Belkacemi 2012 [23] DEB-AMI	AMI	99	DIOR 2 DEB + cobalt-chromium stent	DES (Taxus Liberté)	12	MI, TVR, death	6	30/50	49/49
Byrne 2013 [33] ISAR-DESIRE 3	DES-ISR	268	DEB(SeQuent Please)	DES (Taxus Liberté)	6	Death, MI, TLR	6–8 (angio), 12–36 (clinical)	139/172	145/168
Chae 2017 [24]	De novo lesions	180	DEB(Sequent® Please) + BMS(Coroflex® Blue)	ZES(Resolute Integrity)	9	TLR, MI, cardiac death	12	74/74	72/72
Clever 2014 [25]	De novo lesions	52	DEB(Braun) + BMS(Coroflex Blue)	SES(Cypher)	9	TLR, MI, ST, death	9	16/27	16/25
Cortese 2010 [37] PICCOLETO	small coronary vessels	60	DIOR 1 DEB	DES (Taxus Liberté)	1 after DEB, 3 after BMS, 12 after DES	Death, STEMI, TLR	6 (angio), 9(clinical)	7/28	25/29
Herdeg 2009 [26]	De novo stenoses	136	BMS (Multi-Link Zeta, Guidant)followed by catheter-based local delivery of fluid paclitaxel	DES (Taxus Liberté)	6	TLR, MI, subacute closure, or death	6	NA	NA
Latib 2012 [38] BELLO	Small Coronary Vessels	182	IN.PACT Falcon DEB	DES (Taxus Liberté)	1 after DEB, 12 after DES	Death, MI,TVR	6 (angio), 6–36 (clinical)	91/94	81/98
Liistro 2013 [27]	de novo coronary stenosis	125	Elutax PEB + cobalt-chromium	EES(XIENCE Prime)	3 after DEB, 12 after DES	MI, death, TLR	9	NA	NA
Minguez 2014 [41] BABILON	de novo bifurcated coronary lesions	108	DEB(SeQuent Please) + cobalt-Chromium stent(Coroflex Blue)	EES(XIENCE Prime)	3 after DEB, 12 after DES	Death, MI, TLR	9(angio), 24 (clinical)	43/43	43/43
Pleva 2016 [34]	BMS-ISR	136	PEB(SeQuent Please)	EES(Promus Element)	3 after DEB, 6–12 after DES	MI, TVR, Cardiac death	12	69/69	68/68
Poerner 2014 [28]	coronary artery disease	90	DEB(SeQuent Please) + cobalt-chromium stent(Coroflex Blue)	EES(XIENCE Prime)	NA	NA	6	NA	NA
Stella 2012 [40]	coronary bifurcation lesions	80	Dior I DEB + Liberte BMS	DES (Taxus Liberté)	3 after DEB, 12 after DES	MI, TVR, Death	12	33/33	37/37
Unverdorben 2009 [35] PEPCAD II	ISR	131	PEB(SeQuent Please)	DES (Taxus Liberté)	3 after DEB, 6 after DES	MI, TLR, Cardiac Death, Stent thrombosis	6 (angio), 12–36 (clinical)	66/66	65/65
Xu 2014 [36] PEPCAD China ISR	DES-ISR	215	PEB(SeQuent Please)	DES (Taxus Liberté)	12	MI, TLR, Cardiac death	9 (angio), 12 (clinical)	112/113	107/108
Zurakowski 2015 [29]	de novo coronary lesions	202	DEB(Sequent Please) + BMS	DES(Coroflex Please)	12	MI, TVR, Cardiac death	9	NA	NA

### Primary endpoints

#### Mace

**Fig. 3:**
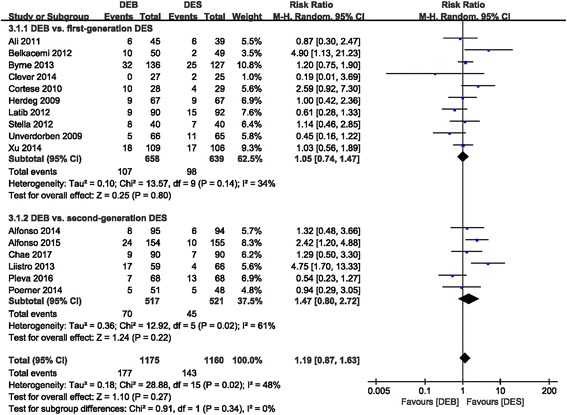
Forest plot of MACE. 3.1.1: DEB vs. first-generation DES; 3.1.2: DEB vs. second-generation DES

**Fig. 4:**
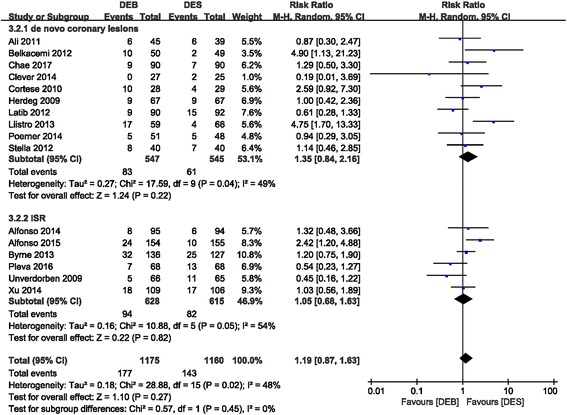
Forest plot of MACE. 3.2.1: de novo coronary disease; 3.2.2: ISR

There was no statistical difference in MACE between the DEB and DES groups (RR 1.19 (0.87, 1.63); *P* = 0.27, I^2^ = 48%). Comparable results were also shown in the DEB subgroup compared to the first-generation DES (RR 1.05 (0.74, 1.47); *P* = 0.80, I^2^ = 34%) and DEB versus the second-generation DES group (RR 1.47 (0.80, 2.72); *P* = 0.22, I^2^ = 61%) (Fig. 3). Another subgroup analysis of DEB versus DES in the treatment of de novo coronary diseases also showed no favorable MACE profile in the DES group (RR 1.35 (0.84, 2.16); P = 0.22, I^2^ = 49%). In addition, no difference in the ISR subgroup (RR 1.05 (0.68, 1.63); *P* = 0.82, I^2^ = 54%) was observed between the DEB and DES groups (Fig. 4).

### Secondary endpoints

#### LLL (in-lesion)

**Fig. 5:**
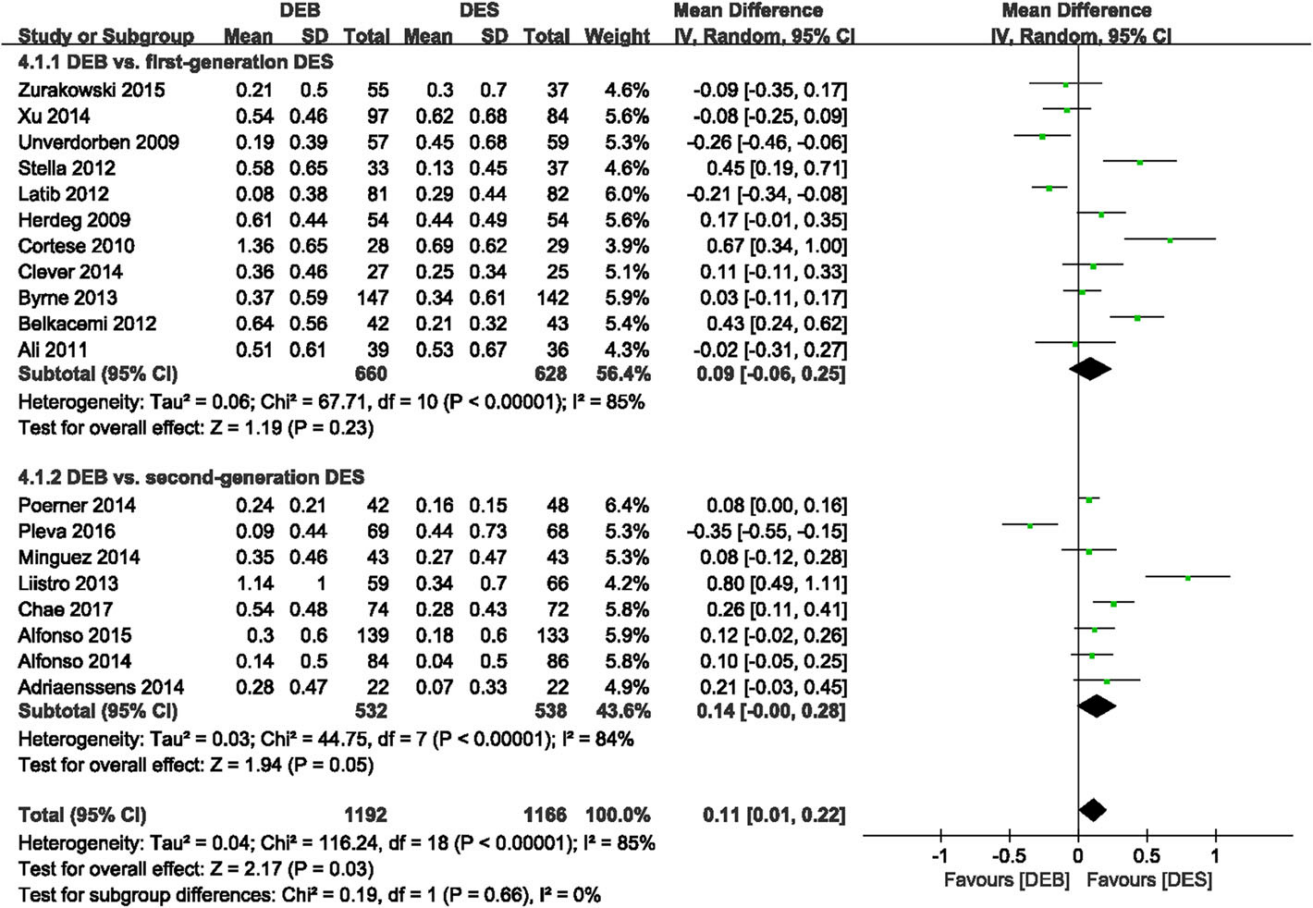
Forest plot of LLL (in-lesion). 4.1.1: DEB vs. first-generation DES; 4.1.2: DEB vs. second-generation DES

**Fig. 6:**
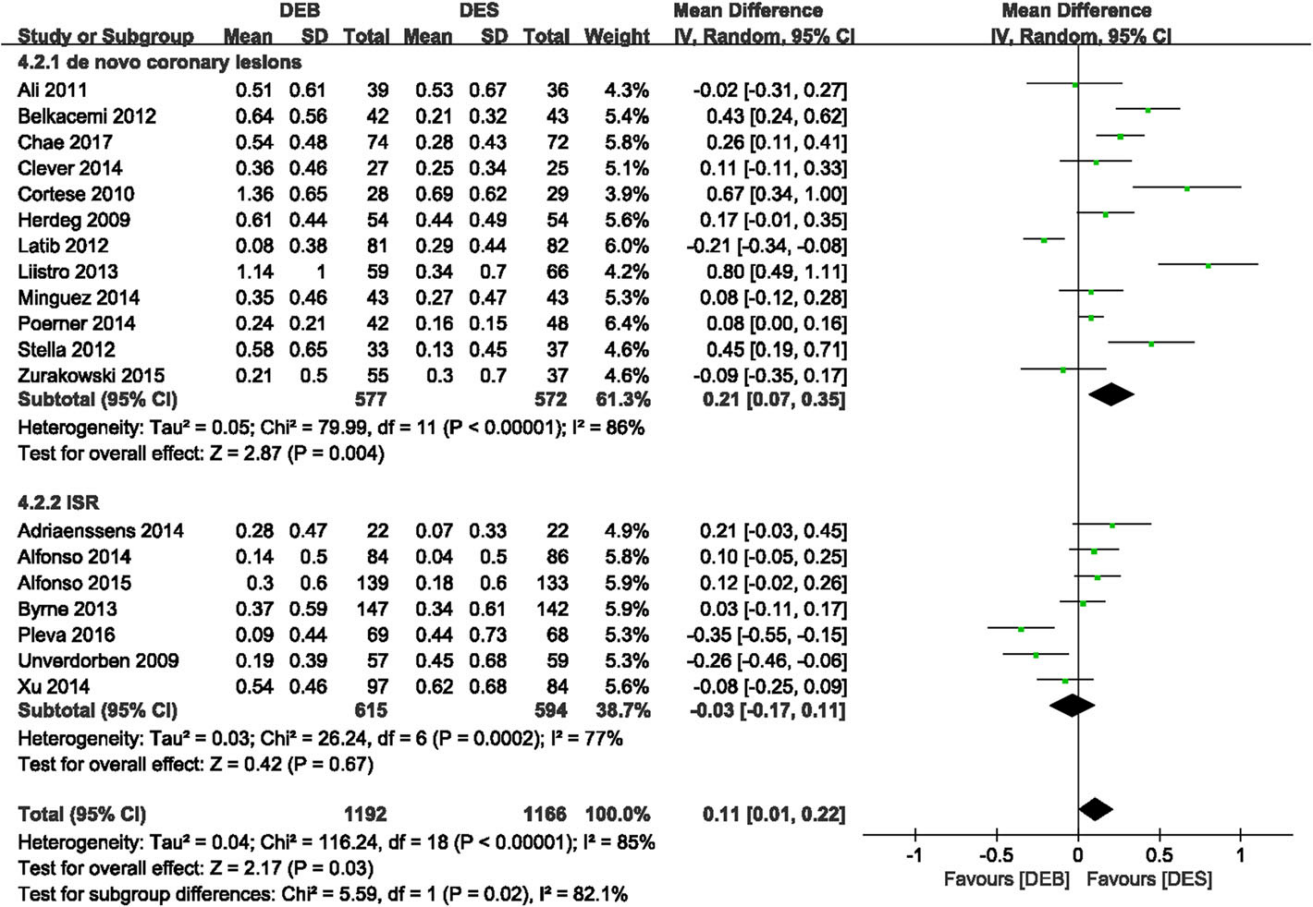
Forest plot of LLL (in-lesion). 4.2.1: de novo coronary disease; 4.2.2: ISR

Compared to the DES group, the DEB group had a significantly larger LLL (MD 0.11 (0.01, 0.22); *P* = 0.03, I^2^ = 85%) (Fig. 5). However, there was no difference between the DEB group and the first-generation DES group (MD 0.09 (− 0.06, 0.25); *P* = 0.23, I^2^ = 85%). In addition, a subgroup analysis of DEB versus DES in de novo coronary diseases showed significant superiority of DES in LLL reduction, with high statistical heterogeneity across trials (MD 0.21 (0.07, 0.35); *P* = 0.004, I^2^ = 86%). Nevertheless, DEB presented similar efficacy in terms of ISR subgroup compared to the DES group (MD -0.03 (− 0.17, 0.11); *P* = 0.67, I^2^ = 77%) (Fig. 6).

#### Br

**Fig. 7:**
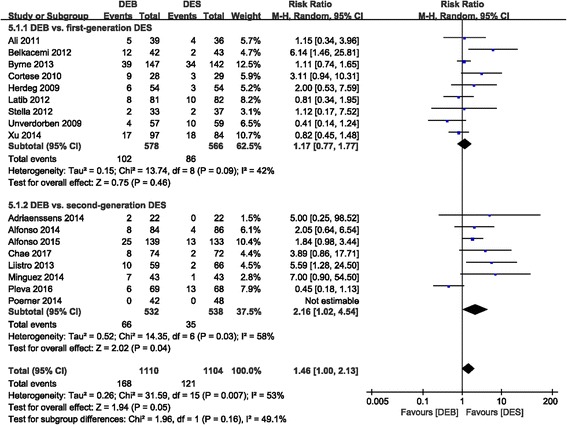
Forest plot of binary restenosis

The difference between the DEB and DES groups was statistically significant with regards to the incidence of BR (RR 1.46 (1.00, 2.13); *P* = 0.05, I^2^ = 53%). The second-generation DES group was significantly different compared to the DEB group (RR 2.16 (1.02, 4.54); *P* = 0.04, I^2^ = 58%). However, in the first-generation DES group, the incidence of BR was similar to that of the DEB group (RR 1.17 (0.77, 1.77); *P* = 0.46, I^2^ = 42%) (Fig. 7).

#### TLR

**Fig. 8:**
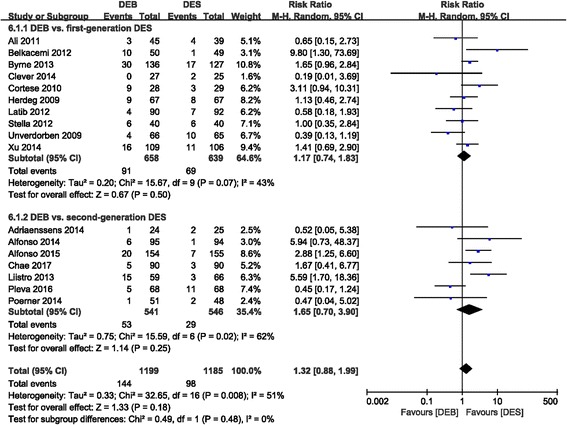
Forest plot of TLR

There was no significant difference in TLR between the DEB and DES groups, with moderate statistical heterogeneity (RR 1.32 (0.88, 1.99); *P* = 0.18, I^2^ = 51%). The TLR occurrence rate in the first-generation DES group (RR 1.17 (0.74, 1.83); *P* = 0.50, I^2^ = 43%) and second-generation DES group (RR 1.65 (0.70, 3.90); *P* = 0.25, I^2^ = 62%) were both comparable to that of the DEB group (Fig. 8).

#### MI

**Fig. 9:**
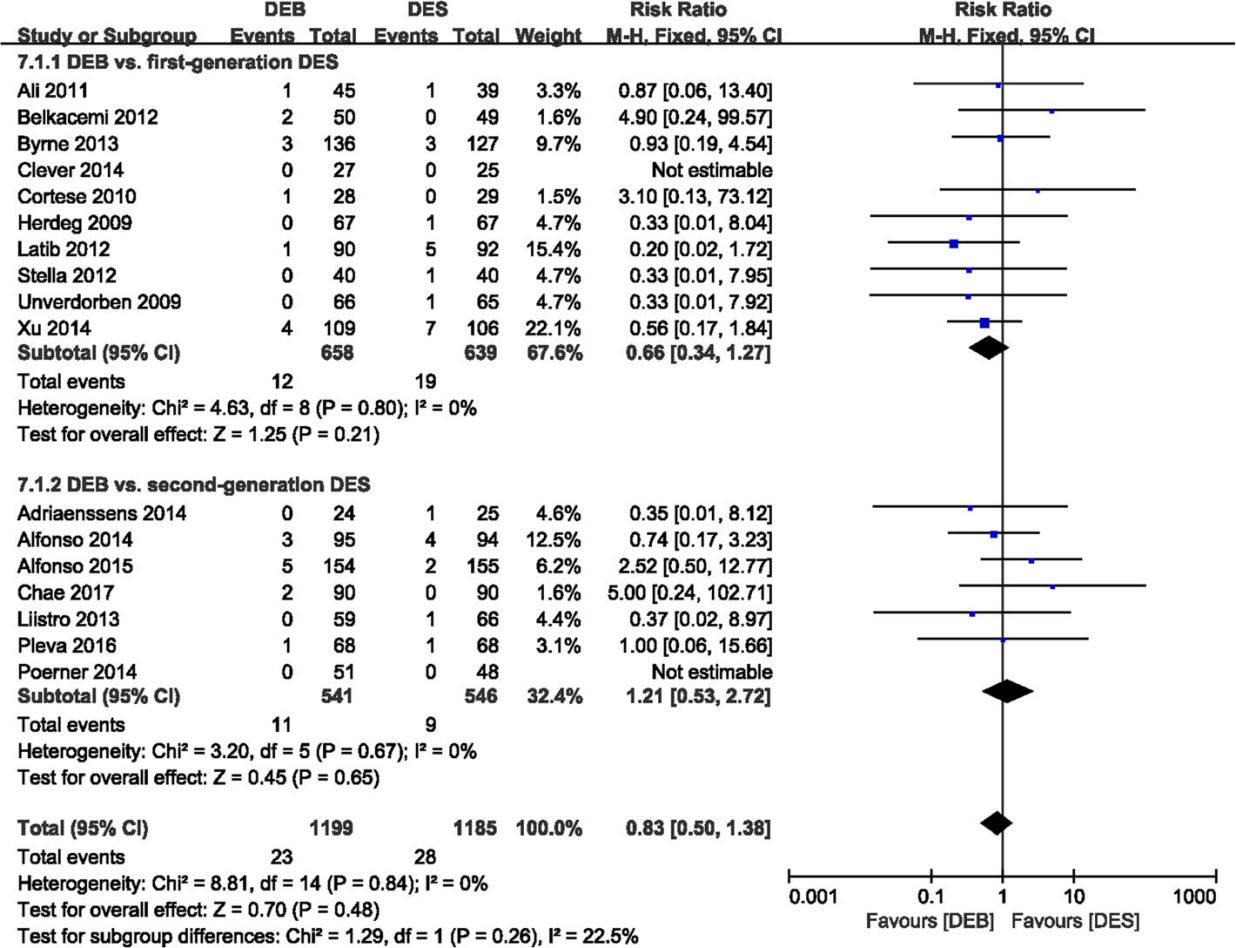
Forest plot of MI

Overall, the risk for MI was not significantly different between the DEB group and the control group, with no statistical heterogeneity (RR 0.83 (0.50, 1.38); *P* = 0.48, I^2^ = 0%), as was also true in the subgroup analysis of the first-generation DES group (RR 0.66 (0.34, 1.27); *P* = 0.21, I^2^ = 0%) and the second-generation DES group (RR 1.21 (0.53, 2.72); *P* = 0.65, I^2^ = 0%) when compared to the DEB group (Fig. 9).

#### Mortality

**Fig. 10:**
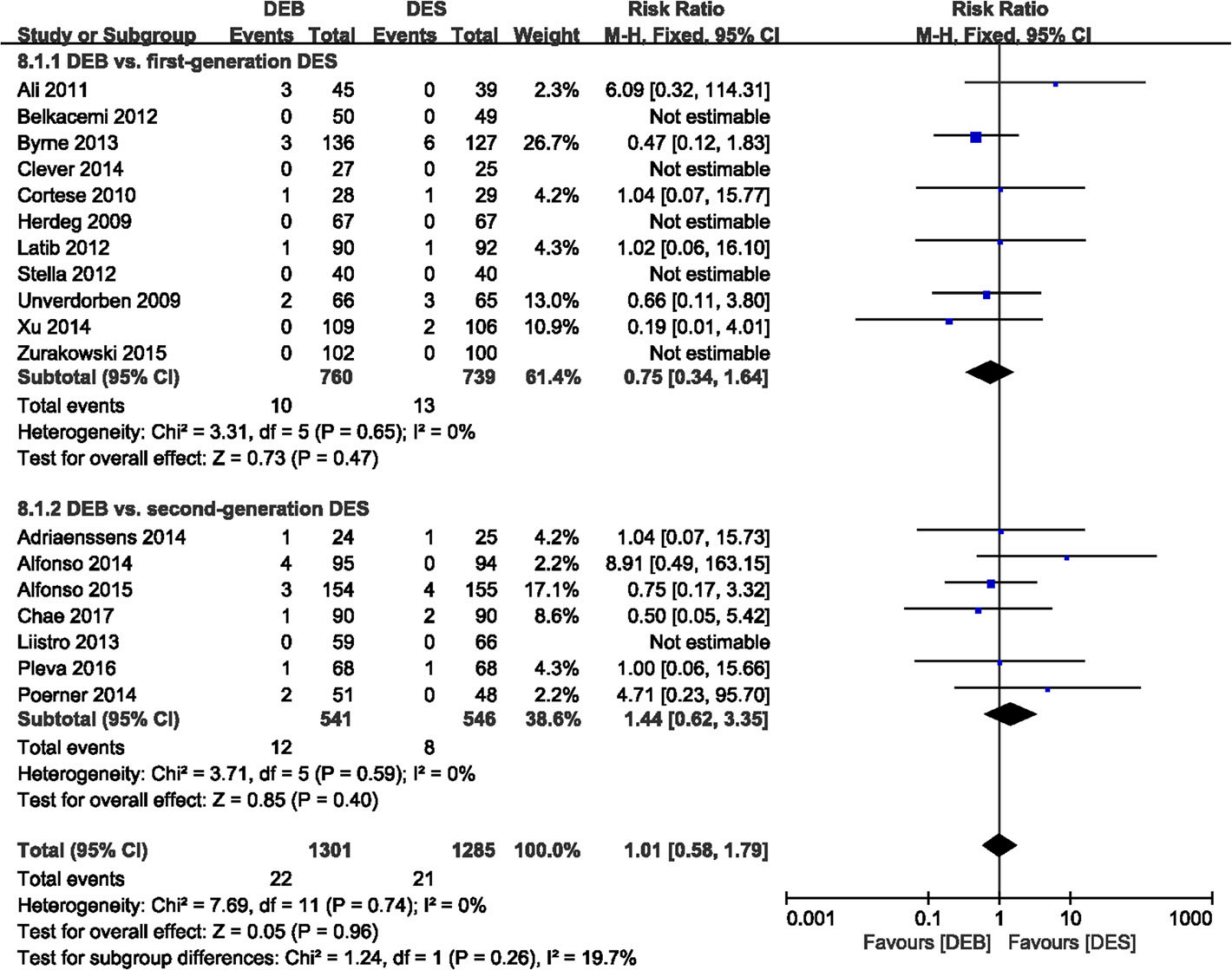
Forest plot of mortality

The mortality rate was not significantly different between the DEB and control groups (RR 1.01 (0.58, 1.79); *P* = 0.96; I^2^ = 0%). Comparable results were also obtained in the subgroup analysis of the first-generation DES group (0.75 (0.34, 1.64); *P* = 0.47, I^2^ = 0%) and the second-generation DES group (RR 1.44 (0.62, 3.35); *P* = 0.40, I^2^ = 0%) compared to the DEB group, respectively (Fig. 10).

#### Long-term follow-up

**Fig. 11:**
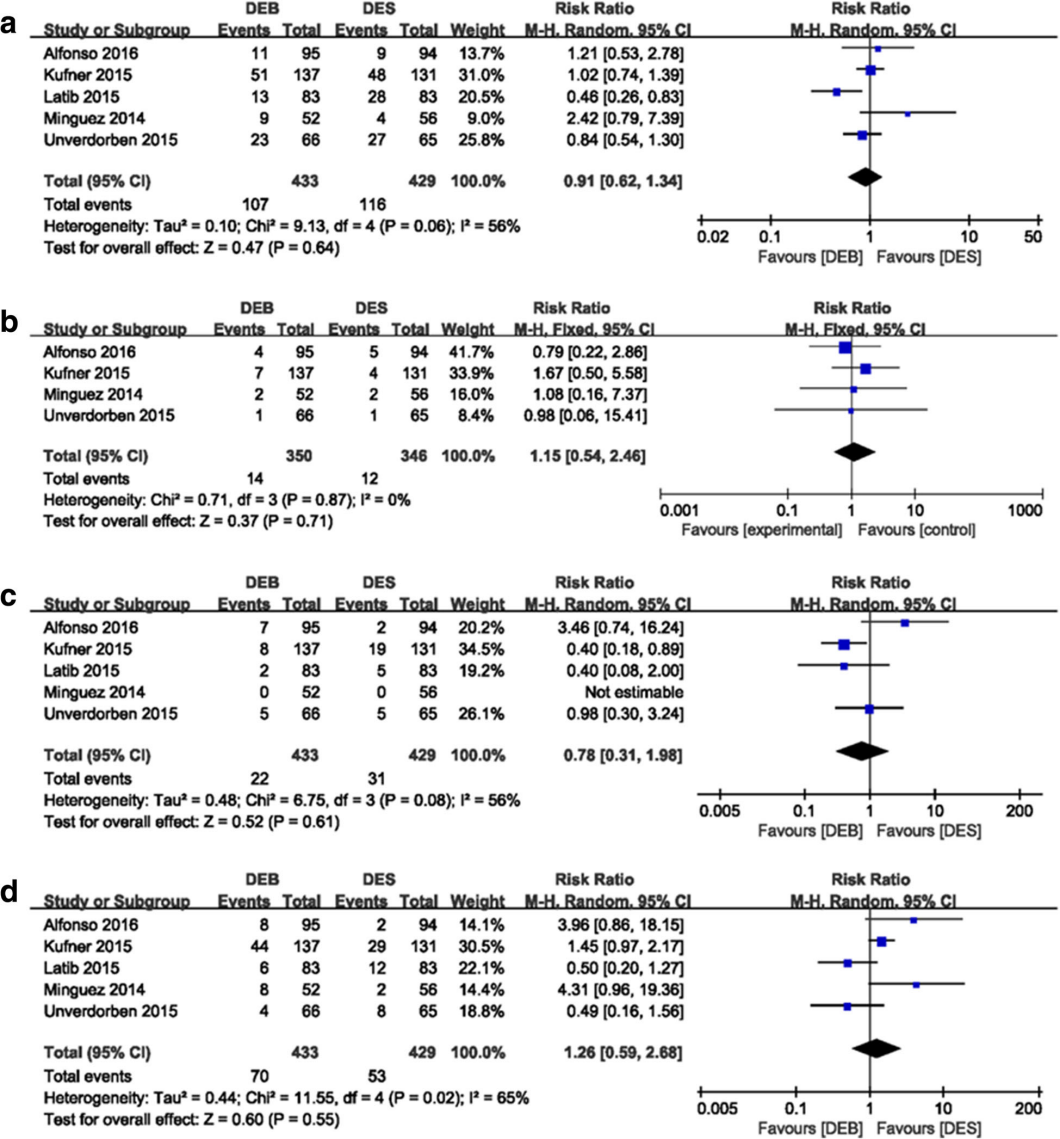
Forest plot of long-term follow-up. **a** MACE; **b** MI; **c** Mortality; **d**: TLR

PEPCAD II, BELLO, ISAR-DESIRE 3, and RIBS V were published in the third year of follow-up, while Minguez’ study [41] was published in the second year of clinical follow-up. Thus, we extracted and conducted the analysis of the meaningful data such as MACE, TLR, MI, and mortality of these studies, and the results showed that the long-term safety was comparable between the DEB and DES groups: MACE (RR 0.91 (0.62, 1.34); *P* = 0.64, I^2^ = 56%); MI (RR 1.15 (0.54, 2.46); *P* = 0.71, I^2^ = 0%); mortality (RR 0.78 (0.31, 1.98); *P* = 0.61, I^2^ = 56%); and TLR (RR 1.26 (0.59, 2.68); *P* = 0.55, I^2^ = 65%) (Fig. 11).

### Sensitivity analysis

After eliminating the included trials one by one, the results did not significantly change when the effect size of each endpoint was pooled together.

### Publication bias test

**Fig. 12:**
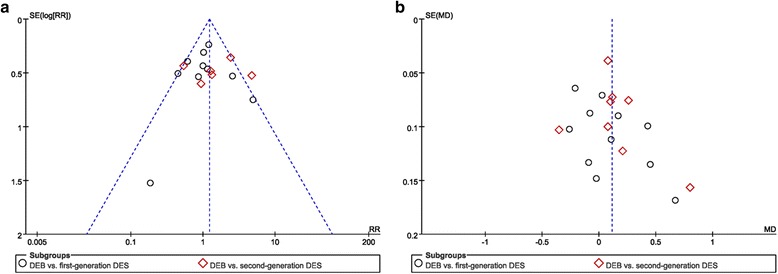
Funnel plot of publication bias. A: MACE; B: LLL (in-lesion)

The Egger test showed no evidence of significant publication bias in the present meta-analysis, according to LLL (in-lesion) (*P* = 0.122) and MACE (*P* = 0.991). The funnel plots of the primary endpoints were symmetrical, further suggesting that there was no publication bias in this meta-analysis (Fig. 12).

## Discussion

A major goal of this meta-analysis was to evaluate the safety and efficacy of DEB (+/-BMS) for treatment of all types of CAD. The main findings of this meta-analysis are summarized as follows: 1) Overall, DEB demonstrated a similar safety profile in the primary outcome of reducing MACE risk and other clinical outcomes, including TLR, MI and mortality, compared to the DES group (regardless of first-generation or second-generation DES); 2) There was a favorable efficacy of reducing LLL and BR at 6 to 12 months angiographic follow-up shown in the DES group, especially in the second-generation DES subgroup; 3) DES was more efficacious in treating de novo coronary diseases, but DES exhibited equivalent efficacy in the treatment of ISR compared to DEB; 4) Analysis of long-term follow-up showed no significant difference between DEB and DES when considering MACE, MI, mortality, and TLR. The present study suggests that DEB may be as efficacious and safe as the first-generation DES, and DEB may also be beneficial with regards to safety concerns compared to the second-generation DES. However, larger trials are needed to confirm these findings.

Despite the significant advances in DES treatment of CAD, some complex vessel lesions such as SVD and bifurcation lesions, which account for 15% - 18% of CAD, are usually accompanied by a high incidence of ISR and often lead to a high probability of revascularization. Many patients with CAD have diabetes, and the vessel lesions in these patients are usually widespread and extensive. In terms of ISR, DES adds an extra layer of struts that could narrow the lumen, and may complicate further development of ISR. However, the Treatment of In-Stent Restenosis by Paclitaxel-Coated Balloon Catheters (PACCOCATH ISR) study, which was the first to apply DEB in humans for ISR treatment [46], showed that DEB was significantly better at reducing ISR compared to the plain old balloon angioplasty (POBA), indicating that DEB could be effective at treating CAD. Due to its highly lipophilic property, paclitaxel is currently widely used in DEB for percutaneous coronary intervention (PCI) [14]. DEB can deliver higher paclitaxel doses (300 to 600 μg) compared to DES struts (100 to 200 μg), the latter of which commonly covers only 20% of the injured vessel wall. Therefore, a larger DEB surface area guarantees more uniform drug delivery to the vascular wall [47]. Recent studies suggested that DEB improved coronary blood supply and vascular function when used to treat severe CAD [48, 49]. Moreover, DEB is more advantageous than DES in terms of homogenous drug application, extensive contact area, absence of stent strut, reducing inflammation and risk of thrombosis, and shortening endothelial healing time [50].

Previous studies have presented inconsistent conclusions regarding DEB compared to DES for coronary artery lesions [47, 51–53]. The present meta-analysis showed that the efficacy of DEB was inferior to DES. However, the diabetic coronary stenosis in Ali’s study [39] and the de novo coronary stenosis in Zurakowski’s study [29] were not statistically different with regards to clinical and angiographic outcomes after 9 months of treatment. Furthermore, Latib et al. [38] reported that that DEB was better than DES for treatment of SVD patients with diabetes, and that DEB generally obtained similar favorable therapeutic effects on small vessel lesions compared to DES. Sinaga et al. [48] also reported that DEB-only angioplasty delivered good clinical outcomes after 1 year of treatment, which were comparable with DES-treated SVD patients. In addition, Naganuma’s study [54] retrospectively investigated the safety of DEB compared to the second-generation DES for treating ISR involving bifurcated lesions. This study also showed that DEB may be an acceptable treatment option with similar incidences of MACE in the two groups.

The lack of efficacy may originate from various factors. One possible reason accounting for DES’s superiority over DEB may be that the pre-dilation with a conventional balloon was not sufficient in the DEB group and that only early-generation DEB was used. Theoretically, prior to the use of DEB, non-coated semi-conformable balloon (balloon to vascular ratio of 0.8–1.0, expansion pressure higher than normal) treatment of lesions could facilitate the transfer of drugs from the balloon to the vessel wall [33]. Current guidelines mandate that the stenosis should first be adequately dilated with a normal balloon angioplasty to ensure the deliverability of DEB to the site of the treated segment [39]. Although pre-dilation with a conventional balloon catheter was commonly used prior to DES deployment, pre-dilation before the use of DEB differed among trials and was usually insufficient. It is not clear in our meta-analysis if this difference was due to the method of balloon deployment. More clinical trials with a large cohort are needed to further corroborate this observation.

Another possible explanation for the findings may be the technique and excipient used on the balloon, which failed to guarantee sufficient drug transfer to the vessel wall. A recent study compared various DEBs in a porcine model and showed that drug concentration in the vessel wall was much higher with the use of DIOR-II DEB than the DIOR-I, demonstrating that effective excipients are necessary to accomplish successful balloon facilitated paclitaxel delivery. The present study showed that DEB was as efficacious and safe as the first-generation DES, which led to an assumption that DEB may achieve better results because of improved techniques and excipients, such as employing new biocompatible polymer coatings.

Other justifications of the above findings include: 1) Paclitaxel released by DEB might not be able to supply powerful intimal response because of its single “shot” approach. However, it has been demonstrated that the amount of paclitaxel transferred to the vessel wall was still in a bio-effective range after 7 days following balloon inflation [55]; 2) The experimental group included DEB with BMS, which may lead to a geographical mismatch between the DEB-treated area and BMS implantation. However, stent length was always inferior to the segment treated with DEB, and we did not observe any evidence of geographical mismatch among these studies; 3) There may be higher neointimal hyperplasia in the DEB group. An IVUS substudy of the PEPCAD III [56] confirmed higher neointimal hyperplasia in the DEB + BMS group. However, a DEB-AMI study [23], which conducted OCT analysis, showed a reduction of neointimal hyperplasia in the DEB group. The present study showed no difference in the reduction of clinical events between DEB and DES, which may need to be further validated.

PEPCAD II [42], BELLO [43], ISAR-DESIRE 3 [44], and RIBS V [45] were published in the third year of follow-up, and Minguez’s study reported data at 24 months clinical follow-up. Thus, the results of MACE, TLR, MI, and mortality extracted from the above studies were statistically analyzed. The DEB and DES groups were not significantly different in long-term safety. However, the population included in this meta-analysis was too small to draw a definitive conclusion regarding safety. Thus, there is an imperative need for longer and larger-scale clinical trials to evaluate long-term effects of DEB. The sample size and power of the current analysis were not adequate to evaluate rare outcomes such as death and ST.

Nevertheless, DEB still had the following advantages over DES: (1) avoiding overlap of the 2-layer or even 3-layer stent and thus reducing the negative effects on coronary anatomy; (2) evenly transporting the drug to the vascular wall, subsequently diminishing delayed endothelialization caused by the heterogeneous distribution of the stent strut; (3) having no polymer, thus not inducing late thrombosis; and (4) reducing the duration of DAPT. Therefore, in clinical practice, DEB is an alternative option for treatment of some CAD, such as SVD, anatomical curved vessels, diffuse long lesions, and bifurcation lesions, when DES is not appropriate. Thus, we believe that, if used properly with the correct operation procedures, DEB could be an optimal choice for CAD treatment, especially for patients who require shorter double antiplatelet therapy or have bleeding tendencies. Still, a considerable number of studies are needed to provide solid evidence to support the replacement of DES by DEB in treatment of SVD and other difficult CADs.

### Limitations

Trials that were included in this study compared DEB with DES in a variety of patients with different types of CAD; the high degree of statistical heterogeneity raised the question of suitability in pooling these trial data, and thus our results in the overall CAD group should be interpreted with caution. Also, the types of DEB or DES used in the included trials were inconsistent, which may be a cause of heterogeneity. However, when we carried out sensitivity analysis, the combined effect of the amount showed no significant changes.

### Outlook

First-generation DES was reported in a majority of the trials included in this meta-analysis. However, the new-generation DES, with thin strut stent platforms, increased biocompatibility and durability, or biodegradable polymers and limus-based antiproliferative agents, has higher efficacy and safety compared to old versions of DES, as evidenced by 10–20% reduction in repeat revascularization [57, 58]. Thus, application of new-generation DES in treatment of CAD may obtain better clinical outcomes. Meanwhile, due to good experience of these drugs used in DES technology, the development of limus-based DEB catheters is of great interest. For instance, zotarolimus and sirolimus DEBs have been developed and tested in pigs with peripheral artery diseases, although they have not been investigated in humans [25, 59]. Cremers and colleagues reported encouraging data with a novel zotarolimus-coated balloon [60]. In order to overcome problems caused by the long-term presence of stents in the blood vessels, biodegradable biological stents (bioresorbable scaffold, BRS) were designed. However, the thicker platform of these DES stents, ease of breaking, and increased tendency for the coated polymer to cause endothelium inflammatory proliferation have limited the application of BRS [61]. The CENTURY II trial [62] compared the effects of BRS and EES on SVD, and suggested that BRS had effects comparable to those of DES within a 12 month follow-up period. As a result, BRS, which will likely be thinner with future development and have a stronger brace and a faster degradation process, may offer better clinical outcomes. However, the current evidence does not support BRS; available evidence showed increased risk of TLR, MACE, and ST with BRS [63].

## Conclusions

From this meta-analysis, we conclude that DEB is comparable to DES in terms of clinical outcomes, and even offered a comparable or even better outcome compared to DES after a 3-year follow-up period, suggesting that DEB is at least as safe as DES. However, larger RCTs are required to assess hard clinical outcomes, such as death, MI or ST, with adequate power.

## Additional file

Additional file 1: Search strategy: Details of search strategy. (DOCX 24 kb)

Additional file 2: Baseline clinical data: Baseline clinical data of patients in the included studies. (DOCX 23 kb)

## Abbreviations

95% CI: 95% confidence interval
BR: Binary restenosis
CAD: Coronary artery disease
DAPT: Dual anti platelet therapy
DEB: Drug-eluting balloon
DES: Drug-eluting stent
ISR: In-stent restenosis
LLL: in-lesion late lumen loss
MACE: Major adverse cardiac events
MD: Mean Difference
MI: Myocardial infarction
PCI: Percutaneous coronary intervention
POBA: Plain old balloon angioplasty
QCA: Quantitative coronary angiography
RCT: Randomized controlled trial
RR: Risk ratio
ST: Stent thrombosis
SVD: Small vessel disease
TLR: Target lesion revascularization
