# Supplementary material for: Comparison of drug-eluting balloon versus drug-eluting stent for treatment of coronary artery disease: a meta-analysis of randomized controlled trials
Source: BMC Cardiovasc Disord. 2018 Mar 2;18:46. doi: 10.1186/s12872-018-0771-y (PMC5834842; doi:10.1186/s12872-018-0771-y)
Supplement: Supplementary file 1 — Search strategy: Details of search strategy. (DOCX 24 kb) [file 12872_2018_771_MOESM1_ESM.docx]

Pubmed

(((((((drug eluting balloon) OR drug-coated balloon) OR drug coated balloon) OR drug-eluting balloon)) AND (((((((((((Drug Eluting Stents) OR Drug-Eluting Stent) OR Stent, Drug-Eluting) OR Stents, Drug-Eluting) OR Stents, Drug Eluting) OR Drug-Coated Stents) OR Drug Coated Stents) OR Drug-Coated Stent) OR Stent, Drug-Coated) OR Stents, Drug-Coated) OR Stents, Drug Coated))) NOT ((((popliteal) OR femoropopliteal) OR infrapopliteal) OR infrainguinal) 1496

Embase

#1 'drug eluting stent'/exp

#2 'drug eluting stents':ab,ti

#3 'drug eluting stent':ab,ti

#4 'stent, drug-eluting':ab,ti

#5 'stents, drug-eluting':ab,ti

#6 'drug-coated stents':ab,ti

#7 'drug coated stents':ab,ti

#8 'drug-coated stent':ab,ti

#9 'stent, drug-coated':ab,ti

#10 'stents, drug-coated':ab,ti

#11 'stents, drug coated':ab,ti

#12 #1 OR #2 OR #3 OR #4 OR #5 OR #6 OR #7 OR #8 OR #9 OR #10 OR #11

#13 'drug eluting balloon'/exp

#14 'drug eluting balloon':ab,ti

#15 'drug-eluting balloon':ab,ti

#16 'drug-eluting balloons':ab,ti

#17 'drug eluting balloons':ab,ti

#18 'drug coated balloons':ab,ti

#19 'drug coated balloon':ab,ti

#20 #13 OR #14 OR #15 OR #16 OR #17 OR #18 OR #19

#21 #12 OR #20

#22 #21 NOT 'popliteal':ti,ab,kw NOT 'femoropopliteal':ti,ab,kw NOT 'infrapopliteal':ti,ab,kw NOT 'infrainguinal':ti,ab,kw 641

Cochrane library

#1 MeSH descriptor: [Drug-Eluting Stents] explode all trees

#2 drug eluting stent:ti,ab,kw (Word variations have been searched)

#3 stent:ti,ab,kw (Word variations have been searched)

#4 #1 or #2 or #3

#5 drug eluting balloon:ti,ab,kw (Word variations have been searched)

#6 drug-eluting balloon:ti,ab,kw (Word variations have been searched)

#7 drug coated balloon:ti,ab,kw (Word variations have been searched)

#8 #5 or #6 or #7

#9 popliteal:ti,ab,kw (Word variations have been searched)

#10 femoropopliteal:ti,ab,kw (Word variations have been searched)

#11 infrapopliteal:ti,ab,kw (Word variations have been searched)

#12 infrainguinal:ti,ab,kw (Word variations have been searched)

#13 #9 or #10 or #11 or #12

#14 #4 and #8

#15 #14 not #13 876
