# Supplementary material for: Comparison of drug-eluting balloon versus drug-eluting stent for treatment of coronary artery disease: a meta-analysis of randomized controlled trials
Source: BMC Cardiovasc Disord. 2018 Mar 2;18:46. doi: 10.1186/s12872-018-0771-y (PMC5834842; doi:10.1186/s12872-018-0771-y)
Supplement: Supplementary file 2 — Baseline clinical data: Baseline clinical data of patients in the included studies. (DOCX 23 kb) [file 12872_2018_771_MOESM2_ESM.docx]

Additional File2: Baseline clinical data

| Study | Age | | Male | | DM | | Hyperlipidaemia | |
| --- | --- | --- | --- | --- | --- | --- | --- | --- |
|  | DEB | DES | DEB | DES | DEB | DES | DEB | DES |
| Adriaenssens 2014 | 67.6±7.7 | 67.6±7.7 | 18 | 25 | 6 | 1 | 24 | 24 |
| Alfonso 2014 | 67±11 | 67±11 | 82 | 82 | 30 | 19 | 69 | 62 |
| Alfonso 2015 | 66±10 | 66±10 | 127 | 139 | 75 | 66 | 110 | 121 |
| Ali 2011 | 62.9±8.1 | 62.9±8.1 | 31 | 33 | 45 | 39 | 37 | 26 |
| Belkacemi 2012 | 59.9±10.9 | 59.9±10.9 | 42 | 41 | 6 | 3 | 11 | 13 |
| Byrne 2013 | 67.7 | 67.7 | 105 | 88 | 56 | 61 | 108 | 103 |
| Chae 2017 | 61.2±11.1 | 61.2±11.1 | 68 | 63 | 28 | 26 | 15 | 18 |
| Clever 2014 | 62.6±13.2 | 62.6±13.2 | 19 | 20 | 7 | 6 | 23 | 16 |
| Cortese 2010 | 68±9 | 68±9 | 22 | 22 | 13 | 11 | 17 | 1 |
| Herdeg 2009 | 64.8±9.4 | 64.8±9.4 | 53 | 45 | 23 | 26 | 46 | 52 |
| Latib 2012 | 64.8± 8.5 | 64.8± 8.5 | 72 | 71 | 39 | 35 | 71 | 73 |
| Liistro 2013 | 66 ± 11 | 66 ± 11 | 49 | 58 | 7 | 10 | 6 | 10 |
| Mínguez 2014 | 63.9±11.3 | 63.9±11.3 | 33 | 37 | 14 | 20 | 36 | 33 |
| Pleva 2016 | 65.6±10.9 | 65.6±10.9 | 43 | 46 | 17 | 18 | NA | NA |
| Poerner 2014 | 68.9±9.5 | 68.9±9.5 | 36 | 36 | 22 | 25 | 39 | 34 |
| Stella 2012 | 63.3±10.4 | 63.3±10.4 | 25 | 29 | 2 | 5 | 21 | 22 |
| Unverdorben 2009 | 64.6±9.7 | 64.6±9.7 | 48 | 50 | 22 | 17 | 52 | 46 |
| Xu 2014 | 61.8 ±9.3 | 61.8 ±9.3 | 88 | 86 | 44 | 35 | 38 | 35 |
| Zurakowski 2015 | 64.1±8.5 | 64.1±8.5 | 68 | 70 | 25 | 20 | 60 | 48 |

To be continued

| Study | Hypertension | | Smoker | | Prior MI | | Prior PCI | | Prior CABG | |
| --- | --- | --- | --- | --- | --- | --- | --- | --- | --- | --- |
|  | DEB | DES | DEB | DES | DEB | DES | DEB | DES | DEB | DES |
| Adriaenssens 2014 | 16 | 15 | 5 | 3 | 12 | 10 | 100 | 100 | NA | NA |
| Alfonso 2014 | 68 | 68 | 56 | 70 | 57 | 56 | 100 | 100 | 4 | 7 |
| Alfonso 2015 | 110 | 121 | 89 | 87 | 73 | 77 | 100 | 100 | 16 | 17 |
| Ali 2011 | 42 | 29 | 22 | 18 | NA | NA | NA | NA | NA | NA |
| Belkacemi 2012 | 18 | 17 | 29 | 19 | 0 | 1 | 1 | 1 | 0 | 0 |
| Byrne 2013 | 105 | 101 | 19 | 15 | 53 | 50 | 100 | 100 | 15 | 32 |
| Chae 2017 | 25 | 25 | 27 | 19 | 3 | 4 | 5 | 9 | NA | NA |
| Clever 2014 | 23 | 23 | 7 | 7 | NA | NA | NA | NA | NA | NA |
| Cortese 2010 | 21 | 20 | NA | NA | 5 | 6 | 3 | 4 | 3 | 4 |
| Herdeg 2009 | 57 | 60 | 11 | 11 | 25 | 16 | 36 | 24 | 6 | 9 |
| Latib 2012 | 72 | 75 | 15 | 10 | 46 | 33 | 52 | 39 | 9 | 12 |
| Liistro 2013 | 22 | 28 | 15 | 23 | 4 | 6 | NA | NA | NA | NA |
| Mínguez 2014 | 32 | 35 | 25 | 29 | 15 | 12 | 12 | 7 | NA | NA |
| Pleva 2016 | NA | NA | 31 | 29 | 43 | 41 | 100 | 100 | 3 | 6 |
| Poerner 2014 | 51 | 48 | 14 | 18 | 21 | 25 | NA | NA | 2 | 1 |
| Stella 2012 | 22 | 21 | 24 | 24 | 7 | 8 | 13 | 12 | 1 | 2 |
| Unverdorben 2009 | 53 | 54 | 16 | 15 | NA | NA | 100 | 100 | NA | NA |
| Xu 2014 | 78 | 69 | 23 | 27 | 53 | 37 | 100 | 100 | 3 | 0 |
| Zurakowski 2015 | 90 | 79 | 17 | 22 | 35 | 43 | 48 | 56 | 1 | 3 |

DM = diabetes mellitus; MI = myocardial infarction; PCI = percutaneous coronary intervention; CABG = coronary artery bypass grafting.
